# Development and testing of TraumaGameplay: an iterative experimental approach using the trauma film paradigm

**DOI:** 10.1080/20008198.2018.1424447

**Published:** 2018-02-05

**Authors:** Joost Asselbergs, Marit Sijbrandij, Evert Hoogendoorn, Pim Cuijpers, Lara Olie, Kfir Oved, Job Merkies, Tessa Plooijer, Simone Eltink, Heleen Riper

**Affiliations:** ^a^ Faculty of Behavioural and Movement Sciences, Section Clinical Psychology, Vrije Universiteit Amsterdam, Amsterdam, The Netherlands; ^b^ The Netherlands and EMGO Institute for Health Care and Research, VU University Medical Centre, Amsterdam, The Netherlands; ^c^ IJsfontein, Amsterdam, The Netherlands; ^d^ GGZ inGeest, Amsterdam, The Netherlands; ^e^ Health and Life Sciences Faculty, Telepsychiatry Unit, Southern Denmark University, Odense, Denmark

**Keywords:** Serious game, prevention, posttraumatic stress disorder, intrusions, early intervention, mobile application, Juego serio, Prevención, Trastorno de estrés Postraumático, Intrusiones, IntervencióN Rápida, Móvil Aplicación, 严肃游戏, 干预, 创伤后应激障碍, 闯入, 早期干预, 移动, 移动应用, •TraumaGameplay is a serious-gaming app, designed to reduce intrusions shortly after trauma and prevent posttraumatic stress disorder (PTSD).•This study tested whether TraumaGameplay, shortly after a trauma film, reduced intrusions among healthy volunteers.•TraumaGameplay did not reduce intrusion frequency; nor did it reduce memory’s vividness and emotionality.•Further development and testing of gaming apps to prevent intrusions is needed.

## Abstract

**Background**: Vivid trauma-related intrusions are a hallmark symptom of posttraumatic stress disorder (PTSD), and may be involved in its onset. Effective interventions to reduce intrusions and to potentially prevent the onset of subsequent PTSD are scarce. Studies suggest that playing the videogame Tetris, shortly after watching aversive film clips, reduces subsequent intrusions. Other studies have shown that taxing working memory (WM) while retrieving an emotional memory reduces the memory’s vividness and emotionality.

**Objective**: We developed TraumaGameplay (TGP), a gaming app designed to reduce intrusions. This paper describes two successive experiments to determine whether playing TGP without memory retrieval (regular TGP) or TGP with memory retrieval (dual-task TGP) reduces intrusion frequency at one week compared to a no-game control.

**Method**: For both experiments, healthy university students were recruited. Experiment 1: 92 participants were exposed to a trauma film and randomized to (1) *regular TGP1* (*n =* 31), (2) *dual-task TGP1* (*n =* 31) or (3) control (*n =* 30). In experiment 2, 120 healthy students were exposed to a trauma film and randomized to (1) regular TGP2 (*n =* 30), (2) dual-task TGP2 (*n =* 29), (3) recall only (*n =* 31) or (4) control (*n =* 30).

**Results**: We found no significant difference between conditions on the number of intrusions for either playing regular TGP or dual-task TGP in both experiment 1 and experiment 2.

**Conclusion**: Our results could not replicate earlier promising findings from preceding experimental research. Several reasons may underpin this difference ranging from the visuospatial videogame used in our experiments to the method of the experiment to the difficulties of replicability in general.

## Abstract (es)

**Antecedentes:** las intrusiones vívidas relacionadas con el trauma son un síntoma característico del trastorno por estrés postraumático (TEPT) y pueden estar involucradas en su aparición. Hay escasez de intervenciones efectivas para reducir las intrusiones y prevenir potencialmente la aparición de TEPT posterior. Los estudios sugieren que jugar al videojuego Tetris, poco después de ver fragmentos desagradables de películas, reduce las intrusiones posteriores. Otros estudios han demostrado que gravar la memoria de trabajo (WM) mientras se recupera un recuerdo emocional reduce la intensidad y la emotividad del recuerdo.

**Objetivo:** Desarrollamos TraumaGameplay (TGP), una aplicación de juego diseñada para reducir intrusiones. Este artículo describe dos experimentos sucesivos para determinar si jugar al TGP sin recuperación de recuerdos (TGP normal) o TGP con recuperación de recuerdos (TGP de doble tarea) reduce la frecuencia de las intrusiones una semana después en comparación con un grupo control sin juego.

**Método:** Para ambos experimentos, se reclutaron estudiantes universitarios saludables. Experimento 1: Se expuso a 92 participantes a una película de trauma y fueron asignados aleatoriamente a (1) TGP1 normal (*n =* 31), (2) TGP1 de doble tarea (*n =* 31) o (3) control (*n =* 30). En el experimento 2, 120 estudiantes sanos fueron expuestos a una película de trauma y asignados aleatoriamente a (1) TGP2 normal (*n =* 30), (2) TGP2 de doble tarea (*n =* 29), (3) solo recuerdo (*n =* 31) o (4) control (*n =* 30)

**Resultados:** No encontramos diferencias significativas entre las condiciones en el número de intrusiones tanto en la de jugar al TGP normal o al TGP de doble tarea en el experimento 1 y 2.

**Conclusión:** Nuestros resultados no pudieron replicar previos hallazgos prometedores previos de investigaciones experimentales anteriores. Varias razones pueden sustentar esta diferencia, desde el videojuego visuoespacial utilizado en nuestros experimentos hasta el método del experimento o las dificultades de replicabilidad en general.

## Abstract (zh)

**背景:** 逼真的创伤相关闯入是创伤后应激障碍（PTSD）的标记性症状，可能在发病时就已经出现了。可以减少闯入和防止随后患上 PTSD的有效干预还非常缺乏。有研究提出，在观看了厌恶性影片片段后立刻玩视频游戏‘俄罗斯方块’，可以减少随后的闯入症状。另外的研究显示在提取情绪性记忆时加重工作记忆（WM）负担可以减少记忆的逼真度和情绪性。

**目标:** 我们开发了‘创伤游戏机’（TraumaGameplay ，TGP)——一个设计用来减少闯入的游戏 APP。本文描述了两个连续的实验来考察和不玩游戏的控制组相比，玩 TGP 的过程中有或者没有记忆提取（有提取 为普通 TGP，无提取为双任务 TGP）是否可以减少一周后的闯入频率。

**方法:** 招募健康的大学生被试进入两个实验组。实验1中，有92名被试观看创伤电影，并随机分配到以下三组：(1)普通TGP1组(*n =* 31), (2) 双任务TGP1 组(*n =* 31) 或者 (3) 控制组 (*n =* 30)。实验2中，有120名健康学生被试观看创伤电影后，被随机分配到以下三组：(1)普通TGP2组(*n =* 30), (2) 双任务TGP2组 (*n =* 29)，(3) 仅回忆组(*n =* 31) 或者(4)控制组 (*n =* 30)。

**结果:** 我们没有发现不同设置组中闯入数量的显著差异，不论是实验1和2中使用普通的TGP 还是双任务 TGP。

**结论:** 我们结果不能重复前人实验研究的有意义发现。这个差异背后的原因有多种可能

性：可能是我们试验中使用的视觉空间电视游戏，或者是总体上这类实验方法本身就有可重复困难。

## 1. Introduction

Psychological trauma is prevalent, with approximately 80% of all individuals estimated to be exposed to a traumatic event during their lifetime (Breslau, 2009). In the first days after exposure, symptoms of re-experiencing, avoidance and hyperarousal are relatively common. Usually, these symptoms decrease over time (Bisson, Cosgrove, Lewis, & Roberts, 2015); however, in approximately 9% of individuals, symptoms persevere and develop into posttraumatic stress disorder (PTSD; Breslau, 2009). Besides significantly compromising a person’s well-being and impairing psychosocial and occupational functioning (Olatunji, Cisler, & Tolin, 2007), PTSD also places a heavy burden on society (Olesen, Gustavsson, Svensson, Wittchen, & Jönsson, 2012). Although evidence-based interventions for PTSD are available (Bisson et al., 2007), studies examining interventions to prevent PTSD’s onset have not been encouraging, and the majority of these studies have shown that they are ineffective or may even increase PTSD symptoms (e.g. psychological debriefing; Rose, Bisson, Churchill, & Wessely, 2002; Sijbrandij, Olff, Reitsma, Carlier, & Gersons, 2006; but see Rothbaum et al., 2012). Currently, interventions that can be offered to trauma survivors in the immediate aftermath are lacking and there is a high unmet need for effective early interventions that can relieve initial trauma symptoms and reduce PTSD rates (Kearns, Ressler, Zatzick, & Rothbaum, 2012; Qi, Gevonden, & Shalev, 2016). New prevention strategies for PTSD should target the acute phase trauma symptoms that are understood to be involved in PTSD’s development and persistence. Intrusions (e.g. involuntary images of the trauma, flashbacks, nightmares) are prevalent in the first hours and days after the trauma and predict PTSD (Bryant, O’Donnell, Creamer, McFarlane, & Silove, 2011; Kleim, Ehlers, & Glucksman, 2007). Thus, interventions that reduce intrusions soon after trauma are promising candidates to prevent the onset of PTSD.

After an (traumatic) event, the memory trace is assumed to undergo a process of gradual stabilization termed consolidation (e.g. Müller & Pilzecker, 1900; Nadel, Hupbach, Gomez, & Newman-Smith, 2012). This consolidation period takes up to six hours (Nader, 2003) and during this period the memory trace is still labile and vulnerable to interference (Nader, 2003; Walker, Brakefield, Hobson, & Stickgold, 2003). It has been proposed that tasks taxing the working memory (WM) shortly after initial learning (i.e. during memory consolidation) may interfere retroactively with memory consolidation (Wixted, 2004).

Studies with healthy volunteers have repeatedly shown that visuospatial tasks, such as modelling clay into predetermined geometric shapes, visuospatial tapping or the videogame Tetris, performed during exposure to a trauma film, resulted in fewer subsequent intrusions compared to no-task controls (concurrent interference; Bourne, Frasquilho, Roth, & Holmes, 2010; Holmes, Brewin, & Hennessy, 2004; Logan & O’Kearney, 2012). Similar studies have also shown that visuospatial tasks performed soon after film viewing, hence when the memory is not yet consolidated, reduced subsequent intrusions by retroactively interfering with memory consolidation (Deeprose, Zhang, DeJong, Dalgleish, & Holmes, 2012; Holmes, James, Coode-Bate, & Deeprose, 2009; Holmes, James, Kilford, & Deeprose, 2010). The effects of verbal tasks, however, are less robust, with some studies finding an increase in reported intrusions (Bourne et al., 2010; [exp. 2]; Holmes et al., 2004, 2010), whilst other studies report no significant difference (Deeprose et al., 2012; Logan & O’Kearney, 2012) or a decrease in intrusions compared to no-task controls (Krans, Näring, & Becker, 2009).

Another line of research argues that vividness and emotionality of distressing memories can be reduced using dual tasks. When retrieving a distressing memory and simultaneously performing a dual task taxing WM, the memory retrieved is less vivid and less emotional, even upon future recall (Baddeley & Andrade, 2000). Thus, it is assumed that dual tasks interfere with memory reconsolidation. The beneficial effects of dual tasks have been found in healthy volunteers retrieving a negative autobiographical memory when performing eye movements (e.g. Leer, Engelhard, & van den Hout, 2014; van den Hout et al., 2011, 2012), drawing complex figures (Gunter & Bodner, 2008) and mental arithmetic (Engelhard, van den Hout, & Smeets, 2011). In addition, the effectiveness of Eye Movement Desensitization and Reprocessing (EMDR) for PTSD has been explained by WM theory (van den Hout & Engelhard, 2012). It has, however, not yet been established whether dual tasks are also effective to prevent intrusions during consolidation of a newly acquired aversive memory.

Although studies have shown that playing Tetris on a computer or Nintendo DS can be effective in a clinical setting (Horsch et al., 2017; Iyadurai et al., 2017), such equipment is not always readily available (e.g. in developing countries, during a natural disaster). With the growing use of mobile technologies, opportunities for large-scale delivery of early interventions have been widely extended. Smartphones have become near ubiquitous, easily available and offer worldwide distribution possibilities through the App Store and Google Play Store; making it a promising tool to target trauma survivors within hours of the traumatic event. Since Tetris is copyrighted we developed a smartphone-based WM taxing videogame called TraumaGameplay (TGP) aimed to reduce intrusion.

The aim of this study was to develop and pilot test the effects of TGP on reducing intrusions using an iterative approach. We therefore conducted two experiments with the aim to alter the TGP I prototype based on the results of the first experiment. To minimize the risk of developing an intervention that could do harm (e.g. psychological debriefing), we considered that experimental testing with healthy volunteers should precede future validation of TGP in real trauma survivors. In this paper, we describe the results of two successive experiments in healthy volunteers, using the trauma film paradigm (Holmes & Bourne, 2008), to determine whether TGP with or without memory retrieval is effective in: (1) reducing intrusion frequency at one week and (2) decreasing vividness and emotionality ratings of the most aversive film memory directly after the intervention and at one week.

## 2. Experiment 1

### 2.1. Development of TraumaGameplay, prototype 1

We created a requirements document for the first prototype. We identified the following requirements: (1) Since TGP should be played shortly after trauma under elevated levels of distress, it is important that the gameplay is easy to comprehend and master, for instance using just one or two buttons; (2) Continuous WM taxation (minimizing alternation between high action and idle moments to ensure that WM is taxed continuously); (3) An optimal level of WM taxation intervals. We chose to program eight playing intervals of 24 s each, with 10 s pause between the intervals. This was based on a previous study on the effects of eye movements during memory retrieval showing that eight intervals resulted in a greater decrease in vividness and emotionality than four intervals (Leer et al., 2014).

**Figure 1.**
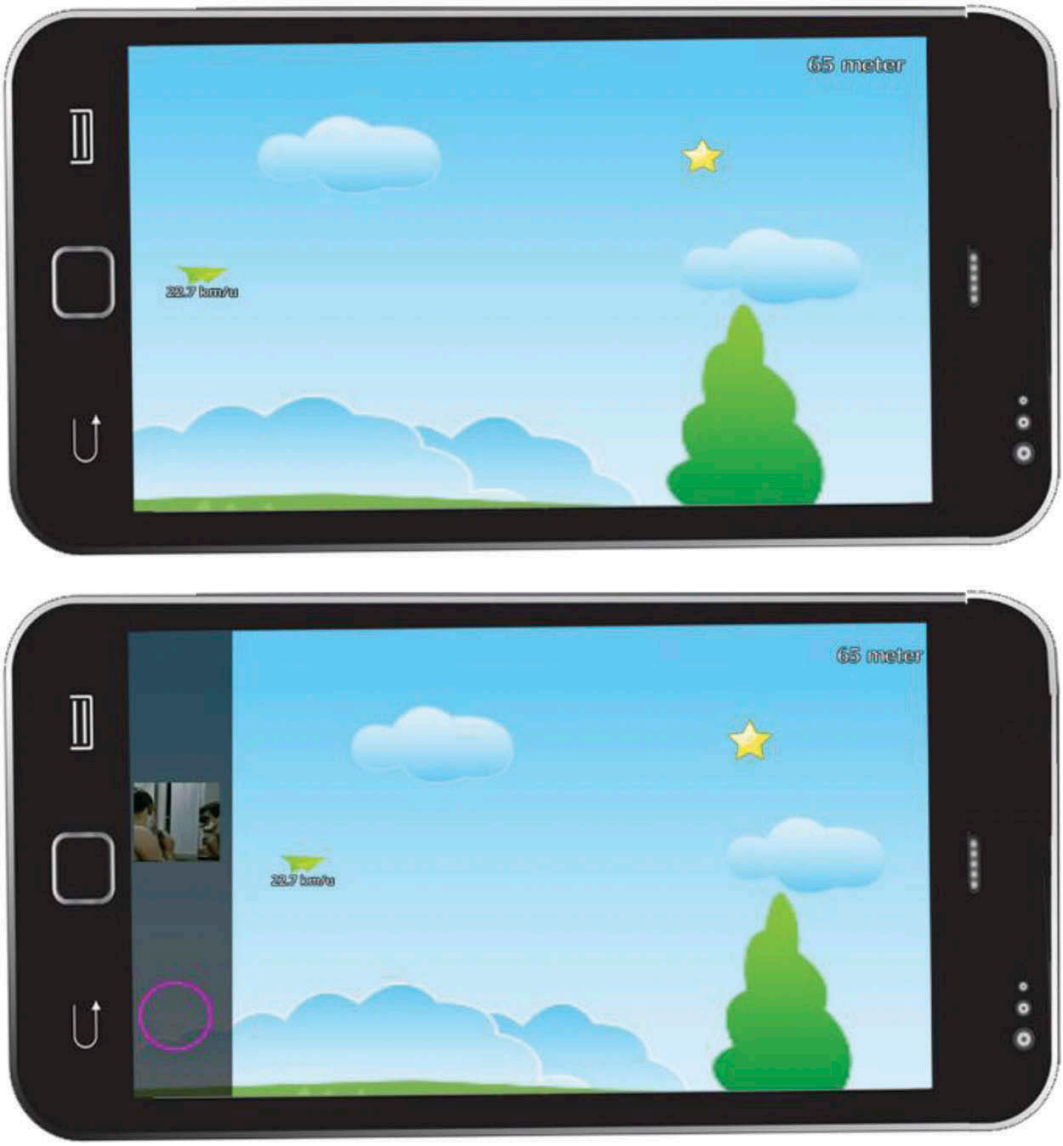
Screenshots of *regular TGP1* (top) and *dual-task TGP1* (bottom).

Based on these requirements we developed two versions: *regular TGP1* and *dual-task TGP1*. *Regular TGP1* is a ‘collect and avoid’ game (Figure 1), where the player guides a paper plane through the air, avoiding clouds and collecting stars. Collecting stars resulted in a speed boost, whereas hitting a cloud resulted in loss of speed. Each level lasted 24 s and the objective of the game was to cover as much distance as possible. *Regular TGP1* had eight levels in total with a 10 s break between levels.

*Dual-task TGP1* resembled *regular TGP1* in terms of gameplay. However, in *dual-task TGP1* participants were instructed, prior to the start of the first level and during each of the subsequent 10 s breaks, to retrieve their most aversive film memory as vividly as possible and keep that memory in their mind whilst playing. Also, at the beginning of the game, participants were shown a grid depicting one image for each scene. These images were taken from a moment just prior to the worst part of the scene (e.g. Holmes et al., 2009; James et al., 2015). The participants then selected the image representing the participant’s most aversive film memory. During each level, the chosen image fell from the top of the screen (on the left side), acting as a reminder cue for the participant to retrieve their most aversive film memory (Figure 1). The image was programmed to appear randomly 12–19 s after the start of each level and it took 3 s for the image to fall from the top of the screen to the bottom. To integrate the reminder image with the gameplay, the paper plane would receive an extra speed boost if the participant pressed the purple circle (Figure 1, bottom left of the screen) when the image and the circle overlapped.

In *dual-task TGP1* the baseline level of complexity was lower than for *regular TGP1*, and the option to manually alter complexity levels was added. This was based on the notion that a dual task should tax WM mildly, so that participants can retrieve the memory while performing the dual task (see Engelhard et al., 2011). Participants were instructed to increase the game complexity whenever they found it too easy to play the game while retrieving the memory, and to decrease the complexity when playing the game and retrieving the memory was perceived as too hard. We programmed four difficulty levels. At difficulty level one, there were no clouds or stars and the participant only had to keep the plane in the air, preventing it from touching either the ground or the top of the screen. Each subsequent difficulty level had more clouds and stars (see Table 1), making it harder to navigate; hence requiring more WM resources to avoid the clouds and collect the stars. To increase/decrease the complexity the participant had to press ‘+’ or ‘-’ at the end of each trial.

**Table 1.** Number of clouds and stars in *regular TGP1* and *dual-task TGP1*.

Difficulty	Number of clouds	Number of stars
Regular TGP1	11	7
Dual-task TGP1		
Level 1	0	0
Level 2	6	0
Level 3	8	4
Level 4	11	7

The aim of experiment 1 was to test the effects of *regular TGP1* and *dual-task TGP1* on intrusion frequency, vividness and emotionality ratings of the most aversive film memory, by comparing *regular TGP1* and *dual-task TGP1* with a no-game control. We predicted that playing *regular TGP1* and *dual-task TGP1* would lead to fewer intrusive memories in the week following the intervention, and lower vividness and emotionality ratings regarding the most aversive memory at post-intervention and at one week, compared to the no-game control.

### 2.2. Method

#### 2.2.1. Participants

Ninety-two students from Vrije Universiteit Amsterdam (VU Amsterdam) were recruited through flyers and Sona Systems (a cloud-based participant pool management solution) to participate in exchange for remuneration (mean age 20.48 years, *SD* = 2.24; 77 female, 15 male). Inclusion criteria: (1) ≥ 18 years, (2) being a student at the VU Amsterdam. Exclusion criteria: symptoms of psychopathology (depression, manic episodes, panic attacks, blood phobia, PTSD), as indicated by the structured Mini International Neuropsychiatric Interview Plus (MINI Plus, Dutch version 5.0.0; Van Vliet & de Beurs, 2007), administrated during session one. All participants signed an informed consent form and approval for the experiment was obtained from the Ethics Committee of the Faculty of Behavioural and Movement Sciences of VU Amsterdam (reference number: VCWE-2014–075).

#### 2.2.2. Materials and measures

##### 2.2.2.1. Demographic questionnaire

Participants were asked to write down their age, sex and ethnicity.

##### 2.2.2.2. Pre- and post-film mood

A composite mood score was calculated by adding up participants’ ratings on two pen-and-paper visual analogue scales (VAS) for ‘sadness’ and ‘anxiety’ on a 100-mm scale that ranged from 0 (not at all) to 100 (extremely).

##### 2.2.2.3. Trauma film

The film (12 min) contained 11 clips of actual/threatened death and serious injury (e.g. a man drowning, people being trampled by an elephant). This film has been used in previous studies as an analogue to real trauma (e.g. Holmes et al., 2009; James et al., 2015). The film was projected on a 180 cm × 240 cm screen using a projector. Viewing distance was 180 cm and the sound was turned on.

##### 2.2.2.4. Thirty-minute filler tasks

After the film, participants rated 14 excerpts of classical music on pleasantness (10 min), then answered non-PTSD related open-ended questions using a personality psychology handbook during the remaining 20 min (based on Holmes et al., 2009, 2010).

##### 2.2.2.5. Visual analogue scale

Pen-and-paper VAS were used to assess vividness and emotionality (van den Hout et al., 2012) of the most aversive memory of the trauma film. Participants were first asked to identify their most aversive film memory and write down three key words on a piece of paper. When administrating the VAS, participants were asked to look at the three keywords and retrieve the memory as vividly as possible and rate the vividness and emotionality of that memory on a 100-mm scale that ranged from 0 (not vivid at all, not at all unpleasant) to 100 (extremely vivid, extremely unpleasant).

##### 2.2.2.6. Film reminder task

The film reminder task consisted of a slide show with 11 neutral stills, representing each of the 11 different film clips. Images were displayed automatically with a 2000 ms interval (Holmes et al., 2009; James et al., 2015).

##### 2.2.2.7. Intrusion diary

Participants kept a pen-and-paper daily diary for seven days, in which they recorded and described each intrusion from the film and whether the intrusion was an image, a thought or a combination of both (e.g. Holmes et al., 2009). Participants were explained (both orally by the research assistant and by written instructions in the diary) that intrusive memories were defined as scenes related to the film that appeared spontaneously in their mind; and that they were not to include memories that they deliberately recalled. Participants were also given a detailed explanation that intrusions can be classified as either an image (e.g. ‘pictures in your mind’s eye’), a thought (e.g. ‘verbal thoughts in the form of words or phrases’) or a combination of both an image and a thought. Intrusion frequency was calculated for both total intrusions and image-based intrusions by respectively counting all intrusion entries and the total number of image-based intrusions per participant (e.g. Holmes et al., 2004, 2009; Homes et al., 2010).

##### 2.2.2.8. Diary compliance questionnaire

Adherence and accuracy in completing the intrusion diary was assessed from 0 (very often/very inaccurate) to 100 (not once/extremely accurate) with the following two VAS: ‘I often failed (or forgot) to write down my intrusions in the diary?’ and ‘How accurate did you fill in the diary?’

#### 2.2.3. Experimental procedure

In the first session, participants’ baseline characteristics were recorded followed by administration of the MINI. Next, participants watched the trauma film, followed by the 30-min filler tasks. Afterwards, the film reminder task was shown to reactivate the film memories and participants were administered the VAS vividness and emotionality (pre-intervention). Next, the participants were randomly allocated to either play *regular TGP1, dual-task TGP1* or the control condition. Participants in the control condition received instructions to sit quietly for 5 min; they were told not to talk to the experimenter and that they could think about anything, without restrictions. After the intervention, the VAS vividness and emotionality (post-intervention) were administrated. At the end of the session, participants received an intrusion diary and were asked to write down all film-related intrusions over the subsequent week. At one week, participants returned their completed intrusion diary and completed the diary compliance questionnaire and the VAS vividness and emotionality (follow-up).

#### 2.2.4. Data analysis

A previous study comparing Tetris to a no-game control found a between-groups effect size of *d *= 0.91 (Holmes et al., 2009). In this study, we aimed to detect a slightly more conservative but still large effect size of 0.8 between *regular TGP1* and *dual-task TGP1* on the one hand and a no-game control on the other hand. Power calculations suggest a minimum sample size of 26 participants per group (power = 0.8, alpha = 0.05, two-sided). Taking into account approximately 15% attrition at follow-up, we aimed to include a total of 90 participants (30 per group).

After the experiment, we discovered that 14 participants in the control condition were erroneously given the added instruction to recall the film clips after watching them.

### 2.3. Results

#### 2.3.1. Baseline characteristics

Table 2 shows participants’ baseline characteristics. The conditions did not differ significantly on sex (*χ^2^*(2, *N *= 92) = 2.085, *p* = .35) and ethnicity (*χ^2^*(2, *N *= 89) = 3.279, *p* = .19); but differed on age (*F*(2, 89) = 5.060, *p* = .008). Post hoc comparisons using the Tukey HSD test indicated that the mean age in *regular TGP1* (*M =* 19.8, *SD =* 1.8) was significantly lower (*p* = 0.08) than in the control condition (*M =* 21.5, *SD =* 2.4). *Dual-task TGP1* (*M =* 20.2, *SD =* 2.2) did not significantly differ from *regular TGP1* or the control condition.

**Table 2.** Means and statistics on baseline and outcome measures (*N =* 92), experiment 1.

Measure	Regular TGP1 (*n =* 31)	Dual-task TGP1 (*n =* 31)	Control (*n =* 30)
Age	19.77 (1.82)	20.23 (2.20)	21.47 (2.24)
Sex (female %)	26 (83.9%)	28 (90.3%)	23 (76.7%)
Ethnicity (Dutch %)	28 (90.3%)	31 (100%)	25 (83.3%)
Mood			
Pre-film	33.58 (27.59)	29.61 (24.08)	41.07 (32.46)
Post-film	66.29 (39.40)	62.71 (39.97)	72.73 (44.38)
Intrusion frequency^a^			
Total intrusions	3.73 (3.42)	3.39 (2.63)	3.87 (3.71)
Image-based intrusions	3.10 (3.21)	2.74 (2.59)	3.00 (2.82)
Diary compliance^a^			
Accuracy	79.17 (12.64)	77.23 (17.14)	80.33 (13.53)
Adherence	11.30 (12.97)	9.74 (19.11)	9.10 (10.63)

Standard deviation or percentages in brackets.

* = *p* < .001.

^a^One participant (*regular TGP1*) reported 22 intrusions, which was over four times the standard deviation. Since this participant did not write down a description for most of the intrusions and was unable to remember their content, we had reason to question the reliability of the entries and excluded this participant from the analysis.

#### 2.3.2. Mood induction

Table 2 shows the pre- and post-film composite mood ratings per condition. A 3 × 2 mixed between-within subjects ANOVA with main factors of condition and time (pre-film, post-film) showed a significant main effect of time on mood (*F*(1, 89) = 91.410, *p* < .001, *η_p_^2^* = .507), indicating that mood deteriorated after film viewing in each condition. There was no significant main effect of condition *F*(2, 89) = 0.917, *p *= .40, *η_p_^2^* = .020 and condition x time *F*(2, 89) = 0.016, *p* = .98, *η_p_^2^* < .001.

#### 2.3.3. Intrusion frequency

Since diary accuracy, diary adherence and intrusion frequency were not normally distributed, non-parametric Kruskal-Wallis tests were conducted. The tests showed that diary accuracy *χ^2^*(2) = 0.261, *p* = .88 and diary adherence *χ^2^*(2) = 1.816, *p* = .40 did not differ significantly between conditions. Also, no significant differences were found between conditions on frequency of both total *χ^2^*(2) = 0.008, *p* = .99 and image-based *χ^2^*(2) = 0.111, *p* = .95 intrusions.

Excluding the 14 control participants who were erroneously instructed to recall the film clips resulted in similar findings for all analyses, i.e. not showing a significant difference between conditions on the frequency of intrusions at one week. Also, treating the 14 control participants as a separate ‘recall only’ condition showed no significant difference.

#### 2.3.4. Vividness and emotionality

**Figure 2.**
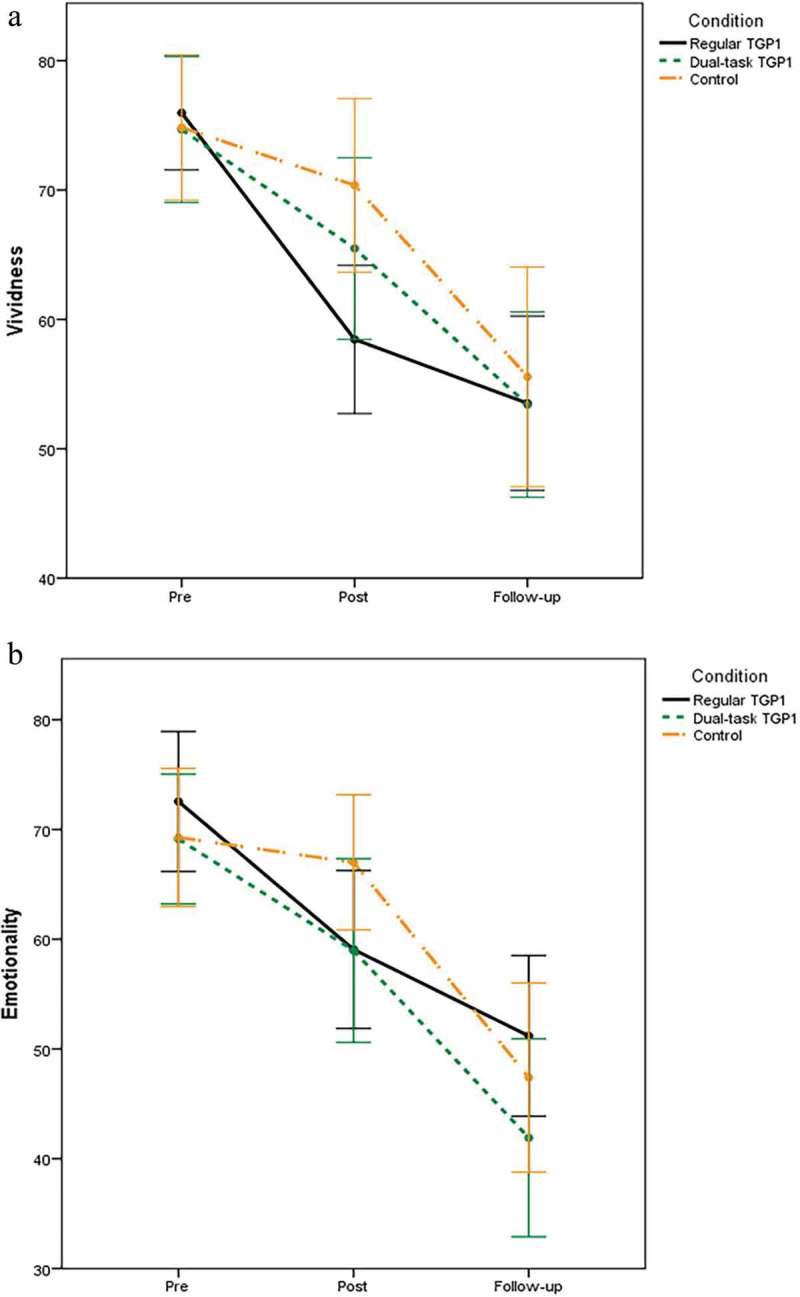
Vividness and emotionality ratings: Experiment 1. Note: error bars represent 95% confidence intervals.

A 3 × 3 mixed between-within subjects ANOVA with main factors of condition and time (pre-intervention, post-intervention, one-week follow-up) showed a significant main effect of time on VAS vividness ratings (*F*(2, 178) = 45.938, *p* < .001, *η_p_^2^* = .340), indicating that vividness decreased over time in each condition (Figure 2a). There was no significant main effect of condition *F*(2, 89) = 0.870, *p *= .42, *η_p_^2^* = .019 and condition x time *F*(4, 178) = 1.669, *p* = .16, *η_p_^2^* = .036.

Regarding VAS emotionality ratings there was a significant main effect of time *F*(2, 178) = 52.269, *p* < .001, *η_p_^2^* = .370, indicating that emotionality decreased over time in each condition (Figure 2b). There was no significant main effect of condition *F*(2, 89) = 0.871, *p* = .42, *η_p_^2^* = .019 and condition x time *F*(4, 178) = 1.681, *p* = .16, *η_p_^2^* = .036 effect.

### 2.4. Discussion: Experiment 1

Contrary to our expectations, we found no significant difference between conditions on the number of total intrusions or image-based intrusion at one week. Also, vividness and emotionality ratings did not differ significantly between conditions, directly after the intervention or at follow-up. A possible explanation why a reduction in intrusion frequency was not observed could be that neither TGP1 versions sufficiently taxed the visuospatial component of WM. For instance, the objective of Tetris, which was found to reduce intrusions in similar studies (e.g. Holmes et al., 2009, 2010), is to manipulate falling blocks by moving them sideways and rotating them by 90-degree units, to create horizontal lines. It is plausible that this kind of gameplay relies more strongly on visuospatial WM resources (e.g. rotating the blocks in the mind’s eye and visualizing the best position to place them); whereas TGP1 (guiding a paper plane through the sky) more likely required good hand–eye coordination rather than tapping into visuospatial resources.

Another possible reason why we may not have detected a significant difference between conditions in terms of number of intrusions at one week is that in our sample the trauma film provoked a low number of intrusions. Our control condition only averaged three intrusions during the week, whereas other studies, using the same film and control condition (e.g. Holmes et al., 2009, 2010), report around seven intrusions. Although some studies have found effects of WM taxing tasks with a low number of intrusions (e.g. Krans et al., 2009; Stuart, Holmes, & Brewin, 2006), it is likely that the lower than anticipated number of intrusions reduced statistical power in our experiment. Also, we did not assess whether *regular TGP1* and *dual-task TGP1* taxed WM to the same extent as Tetris and eye movements, respectively. This may explain why no effect was found on vividness and emotionality ratings and intrusion frequency. It is conceivable that *regular TGP1* taxed WM less than playing Tetris would and consequently failed to successfully interfere with memory consolidation. Conversely, it is possible that WM taxation was higher for playing *dual-task TGP1* compared to eye movements and as a result may prevented participants from keeping the aversive film image in their mind whilst playing *dual-task TGP1*; hence annulling the beneficial effect of the dual task on vividness and emotionality. In experiment 2, these considerations were addressed.

## 3. Experiment 2

### 3.1. Development of TraumaGameplay, prototype 2

**Figure 3.**
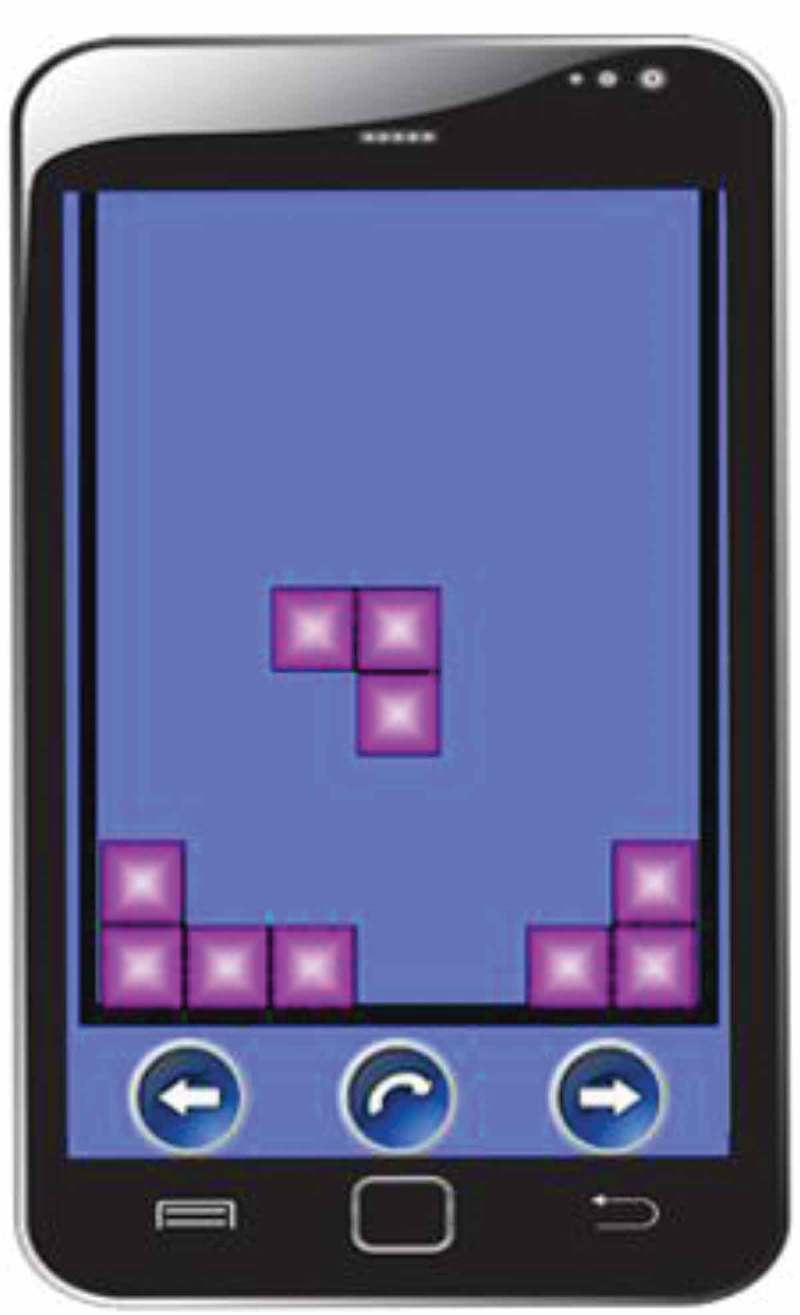
Screenshot of TGP2.

Based on the findings of experiment 1 we developed a new prototype (TGP2) that was more demanding on visuospatial WM than TGP1 and resembled the gameplay of Tetris. Again, we developed two versions. *Regular TGP2*’s gameplay involved rotating and horizontally manoeuvring shapes of three adjacent blocks that fall down from the top of the screen. The objective of the game was to rotate and place the shapes in such a way that they formed 2 × 2 squares (see Figure 3).

*Dual-task TGP2* was identical to *regular TGP2*, except that: (1) the level of difficulty was lower (the blocks dropped at a slower rate), mildly taxing WM in such a way that the participant was still able to retrieve the memory and play the game simultaneously; (2) *regular TGP2* was played uninterruptedly, whereas *dual-task TGP2* consisted of 20 trials, each lasting 24 s with a 10 s break between each trial. Also, compared to *dual-task TGP1*, the neutral film image was not presented during the gameplay and the option to change the difficulty levels was removed, in line with previous protocols used in dual task experiments (e.g. Leer et al., 2014; van den Hout et al., 2012).

Although TGP2 resembles Tetris, there are some differences in gameplay between both videogames, such as: (1) TGP2 used shapes consisting of three adjacent blocks, whereas shapes in Tetris have four adjacent blocks, allowing for more complex shapes; (2) The blocks in TGP2 dropped faster than the blocks in Tetris; (3) The objective in TGP2 is to form 2 × 2 squares, whereas with Tetris the objective is to form horizontal lines; (4) In Tetris a preview of the next shape is shown in the corner of the screen, but TGP2 did not have this feature.

### 3.2. Degree of WM taxation

Prior to the main experiment we tested whether *regular TGP2* and *dual-task TGP2* tax WM enough to deliver the same effects that were found with Tetris and eye movements, respectively. Twenty-four healthy students from the VU Amsterdam (mean age 20.63 years, *SD* = 3.12; 19 female, 5 male) performed a Random Interval Repetition task (RIR; Vandierendonck, de Vooght, & van der Goten, 1998) and were instructed to press the space bar with their non-dominant hand as soon as they heard a beep. The RIR task took 3 min and consisted of 148 stimuli (beeps). Half of the inter-stimulus intervals were 900 ms and the other half were 1500 ms. The array of the inter-stimulus intervals varied quasi-randomly with no more than four successive identical inter-stimulus intervals. The RIR task was administrated in five conditions: *regular TGP2*, Tetris, *dual-task TGP2*, eye movement and no-task control. All participants completed all five conditions, each lasting 3 min. Participants always started with the no-task control. The order of the remaining conditions was counterbalanced. In the no-task condition, only the RIR task was carried out. In the *regular TGP2* condition, participants played *regular TGP2* with a drop speed (the time it takes for a block to drop one row) set to 250 ms. In the *dual-task TGP2* condition, participants played *dual-task TGP2* with a drop speed of 700 ms. In the Tetris condition, the participants played the videogame Tetris Zone (Version 1.2.1; Blue Planet Software, 2007), set to ‘marathon’ mode, on a computer (see Holmes et al., 2009; James et al., 2015). In the eye movement condition, a white dot moved horizontally from side-to-side in the middle of a black background with a speed of 1 s per cycle (left to right to left). The distance to the computer screen was approximately 30 cm and the participants were instructed to keep their head still and follow the dot with their eyes. Since the objective of this experiment was to determine whether *regular TGP2* and *dual-task TGP2* approximated the amount at which respectively Tetris and eye movements tax WM, we predicted that RTs would not differ significantly between *regular TGP*2 and Tetris, and between *dual-task TGP2* and eye movements.

**Figure 4.**
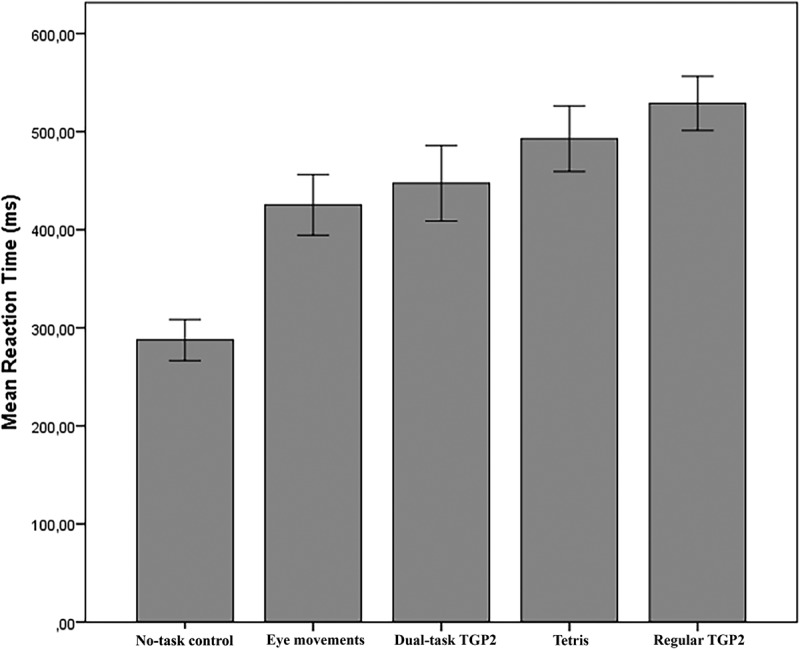
Mean reaction times to beeps during no-task control, eye movements, dual-task TGP2, Tetris and regular TGP2 in a Random Interval Repetition task. Note: error bars represent 95% confidence intervals.

Figure 4 shows the mean RTs for the five conditions. Planned contrasts showed that RTs during *dual-task TGP2* (*M *= 447.36, *SD* = 91.11) did not significantly differ from eye movements (*M *= 425.29, *SD* = 73.30), *t*(23) = 1.527, *p* = .140. However, RTs during *regular TGP2* (*M *= 528.79, *SD* = 65.30) were significantly higher than during Tetris (*M *= 492.78, *SD* = 79.24), *t*(23) = 2.525; *p* = .019. To warrant that *regular TGP2* would tax WM more equally to Tetris, we slightly lowered the drop speed of the blocks in *regular TGP2* to 300 ms.

### 3.3. Method

#### 3.3.1. Participants

One hundred and twenty healthy students (mean age 20.63 years, *SD* = 3.16; 94 female, 26 male) participated in exchange for remuneration or course credits. Inclusion and exclusion criteria were the same as in experiment 1. All participants gave informed consent. Approval for the study was obtained from the Ethics Committee of the Faculty of Behavioural and Movement Sciences of VU Amsterdam (reference number: VCWE-2015–133).

#### 3.3.2. Materials, measures and procedure

Materials and measures were similar to those in experiment 1, except for the trauma film and that participants had to look up answers using a world atlas instead of a psychology handbook during the second part of the 30-min filler task.

##### 3.3.2.1. Trauma film

A new trauma film (12 min) was used, containing seven clips about actual/threatened death and serious injury. Two clips, taken from the film ‘Irreversible’ (directed by Gaspar Noe, 2002), showed a murder in a nightclub and a violent rape. Both scenes have been used previously (e.g. Nixon, Cain, Nehmy, & Seymour, 2009; Verwoerd, Wessel, de Jong, & Nieuwenhuis, 2009). The other five clips were gathered from either youtube (www.youtube.com) or liveleaks (www.liveleaks.com) and involved scenes of eye surgery, an individual set on fire, a car crash aftermath and scenes of assault. We conducted a pilot study which proved that these film clips evoked a sufficient amount of intrusions, similar to the number of intrusions reported by Holmes et al. (2009, 2010).

#### 3.3.3. Experimental procedure

The procedure was similar to experiment 1. Prior to the experimental conditions, participants identified their most aversive memory of the film. Next, participants were randomly allocated to either *regular TGP2, dual-task TGP2*, recall only or control. In the *regular TGP2* condition, participants played *regular TGP2* for 8 min; in the *dual-task TGP2* condition, participants also effectively played for 8 min, dispersed over 20 trials of *dual-task TGP2* each lasting 24 s with a 10 s break between each trial. During this break, participants were instructed to retrieve their most aversive film memory as vividly as possible and keep that memory in their mind whilst playing. The recall only condition was similar to the *dual-task TGP2* condition, except that participants did not play TGP. Participants in the control condition received instructions to sit quietly for 8 min; they were told not to talk to the experimenter and that they could think about anything, without restrictions.

### 3.4. Results

#### 3.4.1. Baseline characteristics

Table 3 shows participants’ baseline characteristics. The conditions did not differ significantly on sex (*χ^2^*(3, *N *= 120) = 3.583, *p* = .31), ethnicity (*χ^2^*(3, *N *= 120) = 3.583, *p* = .31) and age (*F*(3, 116) = 0.660, *p* = .58).

**Table 3.** Means and statistics on baseline and outcome measures (*N =* 120), experiment 2.

Measure	Regular TGP2 (*n =* 30)	Dual-task TGP2 (*n =* 29)	Recall only (*n =* 31)	Control (*n =* 30)
Age	20.03 (2.17)	21.17 (4.23)	20.55 (2.17)	20.77 (3.67)
Sex (female)	24 (80.0%)	20 (69.0%)	25 (80.6%)	25 (80.6%)
Ethnicity (Dutch)	30 (100%)	27 (93.1%)	30 (96.8%)	27 (90.0%)
Mood				
Pre-film	42.37 (27.63)	33.72 (27.69)	44.23 (32.90)	37.57 (25.55)
Post-film	76.97 (35.36)	84.14 (40.27)	76.81 (37.44)	80.83 (40.37)
Intrusions^a^				
Total intrusions	5.79 (4.69)	8.41 (3.96)	6.74 (4.52)	7.03 (5.13)
Image-based intrusions	4.31 (4.08)	6.48 (3.61)	5.03 (3.79)	4.93 (4.09)
Diary compliance^a^				
Accuracy	76.83 (13.86)	74.66 (17.77)	77.06 (16.36)	74.30 (16.49)
Adherence	13.93 (15.82)	16.45 (18.06)	13.90 (20.00)	16.13 (20.94)

Standard deviation or percentages in brackets.

^a^One participant in the regular TGP2 condition reported 30 intrusions, which was over six times the standard deviation, and was excluded from this analysis.

#### 3.4.2. Mood induction

A 4 × 2 mixed between-within subjects ANOVA with main factors of condition and time (pre-film, post-film) showed a significant main effect of time on mood (*F*(1, 116) = 130.056, *p* < .001, *η_p_^2^* = .529), indicating that mood deteriorated after film viewing in each condition. There was no significant main effect of condition *F*(3, 116) = 0.019, *p *= .99, *η_p_^2^* < .001 and condition x time *F*(3, 116) = 1.351, *p* = .26, *η_p_^2^* = .034.

#### 3.4.3. Intrusion frequency

Since diary adherence and intrusion frequency were not normally distributed two non-parametric Kruskal-Wallis tests were conducted. For diary accuracy a one-way ANOVA was conducted. The tests showed that diary accuracy, *F*(3, 115) = 0.235, *p* = .87, and diary adherence, χ^2^(3) = 1.783, *p* = .62, did not differ significantly between conditions. Also, no significant differences were found between conditions on frequency of both total *χ^2^*(3) = 7.41, *p* = .060 and image-based *χ^2^*(3) = 7.66, *p* = .054 intrusions.

#### 3.4.4. Vividness and emotionality

**Figure 5.**
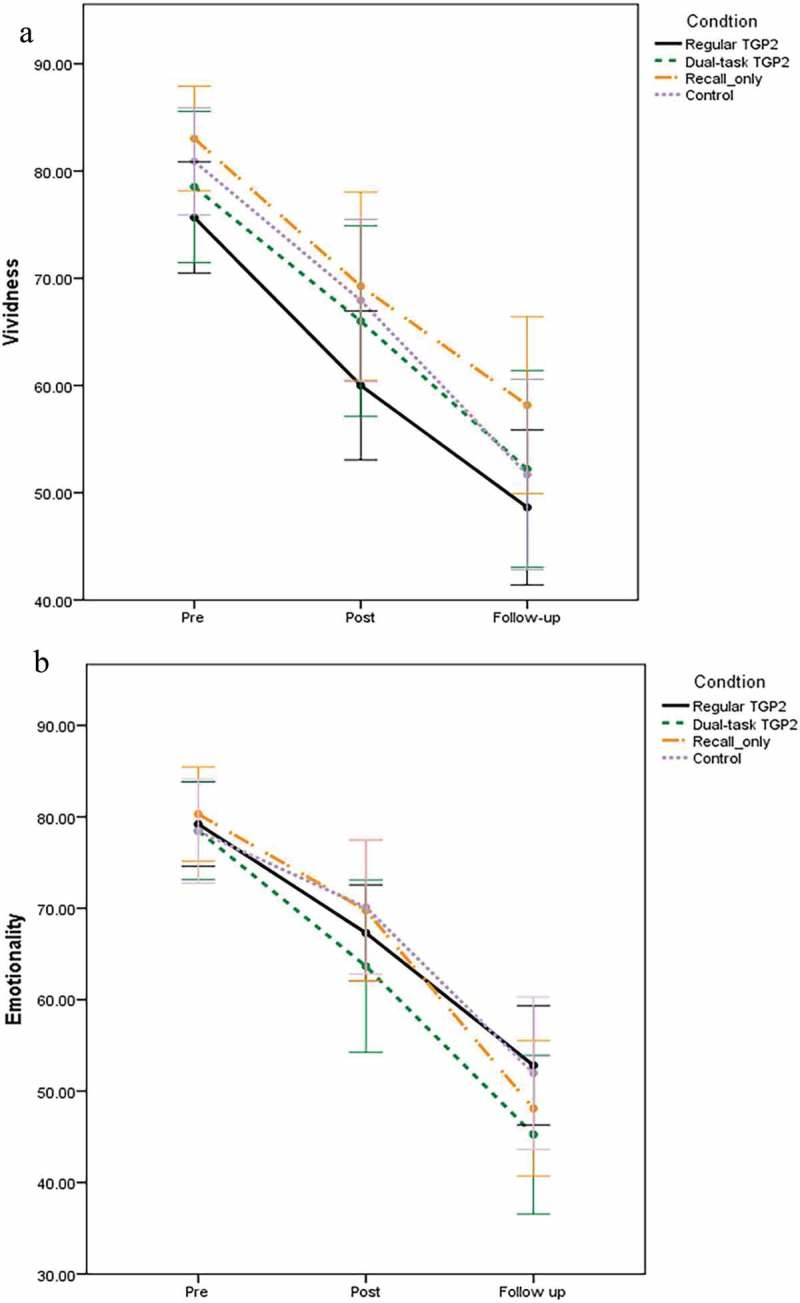
Vividness and emotionality ratings: Experiment 2. Note: error bars represent 95% confidence intervals.

A 4 × 3 mixed between-within subjects ANOVA with main factors of condition and time (pre-intervention, post-intervention, one-week follow-up) showed a significant main effect of time on VAS vividness ratings (*F*(1.77, 203.65) = 104.541, *p* < .001, *η_p_^2^* = .476), indicating that vividness decreased over time in each condition (Figure 5a). There was no significant main effect of condition *F*(3, 115) = 1.512, *p *= .22, *η_p_^2^* = .038 and condition x time *F*(5.31, 203.65) = 0.267, *p* = .94, *η_p_^2^* = .007.

Regarding VAS emotionality ratings, there was a significant main effect of time *F*(1.86, 214.35) = 123.799, *p* < .001, *η_p_^2^* = .518, indicating that emotionality decreased over time in each condition (Figure 5b). There was no significant main effect of condition *F*(3, 115) = 0.597, *p *= .62, *η_p_^2^* = .015 and condition x time *F*(5.59, 214.35) = 0.679, *p* = .66, *η_p_^2^* = .017.

### 3.5. Discussion: Experiment 2

Relative to experiment 1, we made a couple of adjustments as a response to the possible explanations for not finding beneficial effects on intrusion frequency with both TGP1 versions. First, we used a different trauma film. As expected, participants in the control condition reported more intrusions than participants in the control condition of experiment 1. Second, we developed a game that resembled Tetris’ gameplay, to safeguard that TGP2 would tax visuospatial WM. Lastly, we verified that *dual-task TGP2* taxed WM similarly to eye movements and, although we did not test WM taxation of *regular TGP2* after adjusting the drop speed, it is likely that the degree of WM taxation was approximately the same for *regular TGP2* and Tetris.

Again, the results could not support our hypothesis that playing *regular TGP2* or *dual-task TGP2* would decrease the number of intrusions at one week, compared to the control condition. In a similar vein, VAS vividness and emotionality ratings did not differ significantly between conditions, directly after the condition or at follow-up.

## 4. General discussion

This paper described the development and iterative testing of TGP, a gaming app to reduce trauma-related intrusions. We used an iterative approach by conducting experimental research and theory to inform each next iteration of TGP. A set of two experiments was described. Experiment 1 tested the effects of TGP1, a ‘collect and avoid’-type game, in which the player controlled a paper plane with the objective to collect stars and avoid clouds. Experiment 2 tested a second prototype (TGP2), which was a visuospatial gaming app in which the player needed to rotate and place shapes that fell from the top of the screen in such a way that 2 × 2 squares were formed. The main goal of both experiments was to examine whether TGP with and without memory retrieval was effective in reducing intrusions at one week and, secondarily, whether vividness and emotionality ratings of the most aversive film memory decreased post-intervention, compared to controls. Contrary to our expectations, we found no significant difference between conditions on the number of intrusions or the vividness and emotionality ratings in both experiments when compared to controls.

Our finding that *regular TGP1* and *regular TGP2* did not reduce intrusions is at odds with the results of previous studies which have consistently found beneficial effects on intrusion frequency for different visuospatial tasks (e.g. visuospatial tapping, Tetris) performed shortly after watching a trauma film (Deeprose et al., 2012; Holmes et al., 2009, 2010). The reason why this effect was absent in our study is hard to determine. One possibility could be that neither *regular TGP1* nor *regular TGP2* taxed visuospatial WM sufficiently to interfere with memory consolidation. Even though we verified that *regular TGP2* approximated the degree at which Tetris taxed WM, we cannot be completely sure that *regular TGP2* taxed WM enough to sufficiently interfere with memory consolidation. In the studies by Holmes et al. (2009, 2010), Tetris was played on marathon mode, which means that after making 10 lines the difficulty increased. *Regular TGP2*, however, was played on a fixed difficulty, so there is still a possibility that *regular TGP2* taxed WM insufficiently.

Another possibility is that the beneficial effect on intrusions is related to unique features of Tetris’ gameplay. Although *regular TGP2* resembled Tetris, there are some key differences. For instance, Tetris uses a greater variety of different shaped blocks, and shows a preview of the next shape that will appear, which may require different planning and visualization to correctly place the blocks. This, however, seems unlikely, since other visuospatial tasks, such as visuospatial tapping, have also been shown to be successful in reducing intrusions (e.g. Deeprose et al., 2012). Still, it would be interesting for further research to compare different kinds of videogames or apps against Tetris to determine whether the beneficial effects are unique to Tetris or if other videogames are as effective in reducing intrusion frequency.

Our study also showed that *dual-task TGP1* and *dual-task TGP2* did not reduce intrusion frequency compared to controls, which may have been less surprising. To our knowledge, this study is the first of its kind that investigated the effects of dual tasks on intrusions. Previous studies have repeatedly shown positive effects for dual tasks on vividness and emotionality ratings of autobiographic memories (e.g. Gunter & Bodner, 2008; Leer et al., 2014; van den Hout et al., 2011). However, contrary to previous research, neither *dual-task TGP1* nor *dual-task TGP2* reduced vividness and emotionality ratings. Based on the inverted U-curve shaped relationship between emotionality ratings and the degree of WM taxation found by Engelhard et al. (2011), we reasoned that both *dual-task TGP* versions needed to mildly tax WM to facilitate optimal interference with memory consolidation. However, findings from more recent studies contradict the existence of an U-shaped relationship, suggesting a more linear relationship with higher cognitive load increasing the effectiveness of the dual task (van Schie, van Veen, Engelhard, Klugkist, & van den Hout, 2016; van Veen et al., 2015). Also, the effects of dual tasks are typically studied in relation to autobiographic memories, hence memories that have already been consolidated. In our study, a dual task approach was taken to interfere with the consolidation process of not yet consolidated memories. It is possible that dual tasks are not as effective on freshly acquired memories.

Furthermore, it is unclear how the VAS vividness and emotionality relate to overall intrusion frequency, and how administration of these VAS may have influenced the ratings of intrusions frequency. On the VAS emotionality and vividness, participants rated their most aversive memory. It is possible that one scene was selected and rated, but that others still affected the ratings. Moreover, the film scenes that were not targeted for examining vividness and emotionality may still have contributed to the overall intrusions frequency score. Finally, it cannot be ruled out that the administration of the VAS emotionality and vividness may have strengthened the memory for the aversive film clips and thus counteracted the potential beneficial effects of the TGP versions at post-intervention and one-week follow-up.

Limitations of this study were that in experiment 1 the control participants received differential instructions; the trauma film evoked fewer intrusions than expected; and the degree of WM taxation was not measured for *regular TGP1* and *dual-task TGP1*. Experiment 2 tackled these limitations.

Previous positive findings with Tetris have shown great potential for the use of videogames to prevent intrusions. Two recent studies have demonstrated that playing Tetris can effectively reduce intrusions in a clinical setting (Horsch et al., 2017; Iyadurai et al., 2017), highlighting the clinical relevance of playing Tetris shortly after experiencing actual trauma. Our findings are more sobering, and may also reflect a common phenomenon in intervention research, when a new intervention is proposed and tested, with the first studies showing large effects but later studies are much less positive (Ioannidis, 1998). Therefore, replication of initial positive findings remains important and other research groups should be encouraged to replicate the effects of visuospatial videogames on intrusion frequency.

To conclude, our results do not confirm the beneficial effects that have been reported in earlier studies using Tetris or dual tasks. However, our results suggest that promising findings from preceding experimental research may not automatically translate to building and implementing an effective new intervention/app by default. Further development and testing of gaming apps, like TGP, are needed before their clinical effectiveness can be assessed in randomized controlled trials.
